# Sex-Specificity of Oxidative Stress in Newborns Leading to a Personalized Antioxidant Nutritive Strategy

**DOI:** 10.3390/antiox7040049

**Published:** 2018-03-27

**Authors:** Jean-Claude Lavoie, André Tremblay

**Affiliations:** 1 Department of Nutrition, Faculty of Medicine, Université de Montréal, Sainte-Justine Hospital, Montréal, QC H3T 1C5, Canada; 2 Department Obstetrics & Gynecology, and department of Biochemistry and Molecular Medicine, Faculty of Medicine, University of Montreal, Sainte-Justine Hospital, Montréal, QC H3T 1C5, Canada; andre.tremblay@recherche-ste-justine.qc.ca

**Keywords:** sex, gender, oxidative stress marker, newborn, prematurity, glutathione, personalized medicine, antioxidant nutrition

## Abstract

Oxidative stress is a critical process that triggers several diseases observed in premature infants. Growing recognition of the detriment of oxidative stress in newborns warrants the use of an antioxidant strategy that is likely to be nutritional in order to restore redox homeostasis. It appears essential to have a personalized approach that will take into account the age of gestation at birth and the sex of the infant. However, the link between sex and oxidative stress remains unclear. The aim of this study was to find a common denominator explaining the discrepancy between studies related to sex-specific effects of oxidative stress. Results highlight a specificity of sex in the levels of oxidative stress markers linked to the metabolism of glutathione, as measured in the intracellular compartments. Levels of all sex-dependent oxidative stress markers are greater and markers associated to a better antioxidant defense are lower in boys compared to girls during the neonatal period. This sex-specific discrepancy is likely to be related to estrogen metabolism, which is more active in baby-girls and promotes the activation of glutathione metabolism. Conclusion: our observations suggest that nutritive antioxidant strategies need to target glutathione metabolism and, therefore, should be personalized considering, among others, the sex specificity.

## 1. Introduction

Most newborn infants are at risk of oxidative injury following the swift exposure to an oxidant environment at the time of their first breaths, when the partial pressure of oxygen increases suddenly from 35 (umbilical vein) to 85 mmHg (arterial) [1,2,3]. However, this tremendous impulse stimulates several transcription factors (i.e., NFκB and Nrf2) favouring in hours or days, the elevation of endogenous antioxidant defense mechanisms [4,5]. Beyond this stimulation, the general antioxidant capacity [6,7] of preterm newborns remains lower than in term newborns, as suggested by higher levels of oxidative stress markers [8]. The oxidative stress in preterm neonates is frequently explained by a metabolic immaturity or by clinical treatments, such as oxygen supplementation and parenteral nutrition [9]. This situation has been a long-time concern to research teams since this stress is associated with several chronic diseases observed in this population, including bronchopulmonary dysplasia (BPD), necrotizing enterocolitis (NEC), retinopathy of prematurity (ROP) and cerebral haemorrhage [10,11]. Intense research is currently under progress to find ways to prevent oxidative stress in premature infants, and consequently to reduce the incidence of these pathologies. Eventually, an antioxidant therapy easily based on quality of nutrition will emerge. This would be likely performed under a personalized approach according to the age of gestation at birth or to the degree of oxidative stress. Should the sex of the newborn be considered in this definition of personalized antioxidant therapy? It is well known that the sex of the infant is a contributing factor to the incidence of these diseases [12]. For instance, the incidence of BPD [13,14], cerebral palsy and cognitive delay [15], ROP [10,15], among others, are reported to be higher in male infants.

The mathematical logic language suggests that if oxidative stress is at the base of these diseases and that sex of the infant is a contributing factor, then oxidative stress could be different according to the sex (defined here as biological value in contrast to “gender” that has a social value) of the newborn. The confirmation of this mathematical conclusion could influence future clinical personalised practice. Therefore, the purpose of this article is to review what it is known about oxidative stress in the newborn in respect to the sex. On this topic, scientific literature lacks consistency. The discrepancies may arise from the abundance of reported oxidative stress markers as well as biological compartments where they have been measured. The objective was to find a common denominator explaining why some studies document, and others not, differences between sex on oxidative stress markers during neonatal period of infants born at term or preterm.

## 2. Methods

In the present narrative review, literature assessment was undertaken in early 2018 on PubMed. The first search, including the filter “human”, with the following keywords: oxidative stress AND (sex OR gender) AND newborn AND (preterm OR premature OR prematurity), has generated 17 articles. The term “sex or gender” was a great filter since without these two words, the search generated 496 articles. This first assessment, without limit of date of publication, suggested that “oxidative stress” was the first outcome of the investigations, and that the “sex” factor was very secondary. Another reason was that the investigators did not observe differences linked to the sex in their oxidative stress studies and they did not write “sex” or “gender” as keyword.

The search was enriched with the keyword “male OR female” in order to identify studies where the sex of the infants had been monitored. Thus, with the following keywords “oxidative stress AND (sex OR gender OR male OR female) AND newborn AND (preterm OR premature OR prematurity)” the search has generated 275 articles. From them, following reading of title or abstract, 38 were retained for the present article.

## 3. Results

Studies presented in Table 1 have been separated in three sections: studies with no reported comparison between sexes, studies of which the comparison did not reveal a statistical difference, and studies reporting a statistical difference between sexes. For each category, data were subsequently divided according to tissues where markers (of oxidative stress or antioxidant capacity or antioxidant defense) were measured.

A first observation was that difference according to the sex seemed to be linked to the studied tissues. Excepted for F_2_α-Isoprostane of which levels were reported to be sex dependent in two studies out of eight, data suggest that if sex-specific difference exists, it is not found in plasma, urine or bronchoalveolar fluids, which are considered extracellular compartments. In contrast, the difference according to the sex in oxidative stress markers was observed in tissues considered as intracellular compartments, such as erythrocytes, leukocytes, umbilical cord vein and placenta.

For the second observation, Table 1 has been redesigned (Table 2) to present data according to the character of the markers: radical (protein radical injury, lipid radical injury, DNA radical injury, global radical scavenging capacity, uric acid, vitamin A-C-E) or non-radical (glutathione metabolism, cellular uptake of cysteine, superoxide dismutase SOD). Among the 17 radical markers reported, only F_2_α-Isoprostane (reported by two studies out of eight), and protein radical markers and hydroperoxide content in placenta reported by one study, were found as sex-dependent. In contrast, items related to the metabolism of glutathione (glutathione, glutathione peroxidase (GPx), glutathione reductase (GSSG-R), glutathione S-transferase (GST), cellular uptake of cysteine) have been found as sex-dependent, and this in blood cells, placenta and in an umbilical cord vein model.

Finally, a third observation concerns the sex comparison by itself. Levels of all sex-dependent oxidative stress markers are greater and all markers associated to a better antioxidant defense are lower in boys compared to girls during the neonatal period of life.

## 4. Discussion

The principal outcome of the majority if not all assessed articles was to document the presence or not of oxidative stress in the neonatal population, which is not the purpose of the present article. Thus, experiments were not firstly designed to reach a sufficient statistical power to find a sex related difference. So, we must be careful in the fine interpretation of our observations. However, from the point of view of difference according to sex, the clear separation between extracellular and intracellular compartments as well as between radical versus non-radical markers of oxidative stress is remarkable. The few studies of sex-according differences observed in the extracellular compartments can be explained by the fact that these tissues represent a transit reservoir for waste cell products before their elimination, such as metabolites generated after a radical injury. These dynamic changes could not be enough sensitive to observe a difference between sexes. On the other hand, the most frequent observation of sex-according differences was done on glutathione and enzymes or systems in relation to glutathione. Glutathione metabolism is mainly an intracellular process. It controls the intracellular levels of peroxides (via GPx), aldehydes (via GST) and even radicals (via regeneration of oxidized vitamins C and E); thus, changes in glutathione metabolism could also explain the sex-specific differences reported for F_2_α-Isoprostane. The high significance between studies cited here on the difference between sexes suggests a different capacity of newborns to cope with an oxidative environment depending of their sex. Therefore, our suggestion from the mathematical logic conclusion outlined in the Introduction that the “oxidative stress could be different according to the sex” appears to be substantiated. Each data reporting a difference according to sex shows higher levels of oxidative stress markers and a lower antioxidant capacity (related to glutathione metabolism) in boys compared to girls. This observation, in line with the fact that the prevalence of diseases in baby boys is higher than in girls [12], suggest that an improvement of glutathione status could prevent or at least reduce prevalence of these diseases.

Glutathione defense is specialized in peroxide detoxification. Thus, with less effective glutathione metabolism, one can expect that males or cells from male infants are more susceptible to injury by peroxides. As part of the studies undertaken to understand the clinical impact of exposing newborn infants to an oxidant environment (such as the one occurring during specific clinical treatments), one was designed more than twenty years ago to assess the impact of peroxides on endothelial production of eicosanoids [46]; by this time the sex was already suspected to be an active factor. The experimental design was a 3.5 h perfusion of umbilical cord vein (from term infants born after repeated Cesarean sections) with a nutritive solution containing or not (control) organic peroxide (*tert*-butylhydroperoxide). Eluates were collected each hour. The pattern of release of the four eicosanoids and glutathione differed according to whether the umbilical cord vein derived from boys or girls. The glutathione release was not affected by peroxide in veins derived from girls, whereas the veins from boys were losing their glutathione content. This study revealed that the equilibrium between peroxides and glutathione differed between sexes. This difference according to the sex of the infants was also emphasized few years later by the demonstration that a short exposure (3–4 h) to *tert*-butylhydroperoxide induced the mortality of endothelial cells isolated from umbilical cord vein from infants born at term in primary culture; the mortality was higher when cells were derived from boys [6]. These two studies underline the relative fragility to an oxidative environment of cells from boys at time of birth. 

Infant mortality is well known to be higher among boys. For instance, the World Health Organization reported that, in 2010, the prevalence rate was of 48 vs. 45/1000 births (M vs. F) in the world [50]; without China and India, the rates were 52 vs. 44/1000 births (M vs. F) [50]. The difference according to sex seems receded in function of time after birth in term infants. In situation reminiscent to the previous cited studies [6,46] concerning umbilical cord vein endothelial cells exposed to peroxides, infants born before 32 weeks of gestation are also routinely exposed to peroxides during the time of their parenteral nutrition. Indeed, parenteral nutrition is contaminated with high levels of peroxides [43,51,52]. These peroxides are strongly suspected to be at the origin of several diseases observed in this population. A recent meta-analysis [53] compared clinical outcomes of premature newborns according to whether or not their parenteral nutrition formula was photo-protected. Adequate protection against ambient light reduces by half the generation of peroxide in the parenteral nutrition [43,51,52]. The photo protection was associated to a 50% reduction in mortality at 36 weeks post-menstrual age. In girls, the reduction was from 11 to 4% whereas in boys, it was from 20 to 9%; note that the difference according to sex remained.

The literature has abundantly reported a high oxidative stress in preterm infants, and that stress is associated with several pathological complications observed in this population. Although the sex factor is well described for these diseases, the discrepancies between sex and oxidative stress cast doubt on the link between these parameters. Our observation reinforces the concept of the existence of a difference due to the sex of the newborn by specifying the type of markers to be monitored and in which biological compartment does it occur. The fact that the specific marker to follow is linked to glutathione metabolism suggests a role for estrogens to prevent or at least diminish undesired responses to oxidative stress. Indeed, circulating estrogen concentrations are very high during foetal development mostly due to the foetal adrenal contribution of precursor DHEA (dehydroepiandrostenedione) and subsequent placental conversion to estrogen [54]. As such, estrogens are clearly associated with embryogenesis and intrauterine sexual development. In addition, cellular effects to circulating estrogens are mainly dependent on the respective contribution of estrogen receptors ERα and ERβ, of which sex-specific differences in terms of their expression levels were reported [55,56,57]. Estrogen receptors have been shown to directly induce genes associated to cellular responses to oxidative stress, such as glutathione peroxidase, in foetal and adult tissues, strongly supporting a role of estrogen in the protection against oxidative stress [58,59,60]. Hormone activation of estrogen receptors also contributed to enhance the response of transcription factors Nrf2 and NFκB, resulting in induced expression of several genes encoding for antioxidant enzymes involved in glutathione metabolism [61,62,63]. 

Because there is increasing evidence on the importance of a fine regulation of homeostasis between oxidant and antioxidant molecules for a healthy development, the antioxidant strategies must be an essential part of clinical practice. This clinical approach should be personalized according, among others, to the sex of the newborn. Our observations suggest that these strategies should target glutathione metabolism. Certainly, one way to be favoured in the application of a personalized antioxidant strategy is enrichment of mother milk or parenteral nutrition, according to the mode of nutrition needed by the newborn. Human milk is known to have antioxidant properties since oxidative stress markers are lower in infants fed with human milk compared to infant formulas [64]. This property seems to depend, at least in part, from short peptides generated during digestion of milk by the baby. Thus, a promising approach would be to enrich the milk with specific hexapeptides as those generated following mimicking digestion of human milk [65]. These peptides have been demonstrated to have excellent antioxidant properties in vitro [65] and in vivo where they promote a glutathione increase [66]. However, further studies are needed to validate the benefit of such enrichment of milk formulas and possibly of human milk in order to improve antioxidant defense especially in preterm newborns. To reduce the incidence of bronchopulmonary dysplasia by improving glutathione status in premature infants (those on parenteral nutrition), several studies have tested the addition of cysteine (limiting amino acid for synthesis of glutathione) or N-acetylcysteine (precursor of cysteine). A meta-analysis underlined the failure of these approaches as much for glutathione level as for the chronic lung disease [67]. Others have proposed to infuse glutathione to prevent oxidative stress and lung damage in newborn animals exposed to high levels of oxygen [68]. Based on the fact that glutathione by itself has for a long-time been recognized to be a physiological pool of cysteine [69], a recent animal study has considered glutathione as a physiologic precursor of cysteine [70]. The enrichment of the intravenous solution with glutathione prevented the negative impacts of peroxides generated in parenteral nutrition on the oxidative stress, glutathione status, and lung integrity [70].

## 5. Conclusions

The importance of redox biology in health [71,72], as well as the increasing evidence for long-term influence of oxidative stress undergone early in life [73,74], supports the development of antioxidant approaches, especially during the neonatal period. The prevention of oxidative stress at this age is critical to warrant a healthy development throughout the whole life. Hence, the concept developed here strongly suggests that this approach should be personalized by including the sex specificity.

## Figures and Tables

**Table 1:** Studies in function of the type of comparisons reported between sexes.

Tissues: Markers	Studies without Comparison between Sexes	No Statistically Difference between Sexes	Statistically Difference between Sexes	
Plasma/serum:				
F_2α_-isoprostane	[16,17,18]		M > F	[19,20]
MDA/aldehydes	[21,22]	[20]		
TBARS	[23]	[24]		
Hydroperoxides	[25]	[20]		
Protein carbonyl	[18]			
Nitrotyrosine	[18]			
Ascorbyl radical	[26]			
DNA damage	[27]			
TOS	[28]	[24]		
TAC	[28]	[19,29]		
TAOC	[17,30]	[24]		
FRAP		[24]		
Glutathione	[31]			
SOD	[16,30]			
GPx	[30]			
Vitamin C	[32,33]			
Vitamins E, A	[32]			
Erythrocytes/cord blood/peripheral blood:				
TBARS	[23]			
Redox potential of glutathione	[8,9]			
Se		[34]		
GPx	[28]	[34,35]	M < F	[36]
GSSG-R		[35]	M < F	[36]
GST		[35]	M < F	[37]
CuZnSOD		[34]		
Urine:				
F_2γ_-isoprostane	[8,16,38]			
8-OHdG	[38,39,40,41,42]			
Dityrosine	[8]			
Peroxides	[43]			
Bronchoalveolar lavage fluide/tracheobronchial aspirate fluid/airway aspirates:				
MDA	[13]			
Protein carbonyl	[44]			
TAA	[45]			
Vitamin C	[44]			
Acid uric	[44,45]			
Umbilical cord vein:				
Glutathione			^a^ Exposed to tBH: efflux M > F	[46]
Leukocytes isolated from tracheal aspirate:				
Glutathione			M < F	[6]
Glutathione synthesis		[47]		
GSSG-R			Exposed to FiO_2_ > 0.25: M < F	[6]
γ-GT		[47]		
Cysteine uptake			M < F in Pre-term	[48]
Placenta:				
Protein carbonyl			M > F	[49]
Hydroperoxides			M > F	[49]
Nitrotyrosine			M > F	[49]
GPx			M < F	[49]

FRAP: Ferric reducing ability of plasma; GPx: glutathione peroxidase; GSSG-R: Disulfide glutathione reductase; GST: Glutathione S-transferase; γ-GT: gamma-glutamyltranspeptidase; MDA: malondialdehyde; 8-OHdG: 8-hydroydeoxy-guanosine; SOD: superoxide dismutase; TAA: Total antioxidant activity; TAC: Total antioxidant capacity; TAOC: Total antioxidant capacity; TBARS: thiobarbituric acid reactive substance; TOS: Total oxidant status; ^a^ Umbilical cord vein infused with *tert*-butylhydroxyperoxide, efflux of glutathione.

**Table 2:** Studied tissues in function of the type of oxidative stress markers (designed from Table 1).

Oxidative Stress Markers	Studied Tissues, Sex Effect Not Reported	Studied Tissues, Sex Effect Reported	Results
Radical injury/protein:			
Carbonyl	A.F.; ^a^ plasma	placenta	M > F
NitroTyrosine	plasma	placenta	M > F
Dityrosine	urine		
Radical injury/lipid:			
F_2_α-isoprostane	Urine ^b^ (3); plasma (3)	Plasma (2)	M > F
MDA/aldehydes	A.F.; plasma	Plasma	^c^ =
TBARS	A.F.; plasma	Plasma	=
Radical injury/DNA:			
8-OHdG	Urine (5)		
Radical scavenging capacity/global:			
TOS	plasma	Plasma	=
TAC	plasma	Plasma (2)	=
TAOC	plasma	Plasma	=
TAA	A.F.		
FRAP		Plasma	=
Others radical markers:			
Vitamin C	A.F.; plasma		
Vitamin E, A	Plasma (2)		
Ascorbyl radical	Plasma		
Uric acid	A.F. (2)		
Glutathione metabolism:			
Glutathione	Plasma	Umbilical cord vein	^d^ loss: M > F
		Leukocytes	M < F
Redox	^e^ Erythrocyte (2)		
Se		Erythrocyte	=
GPx	Erythrocyte; plasma	Erythrocyte (3);	M < F (2); = (1)
		Placenta	M < F
GSSG-R		Leukocyte	M < F
		Erythrocyte	M < F
GST		Erythrocyte (2)	M < F; =
γ-GT		Leukocyte	=
GSH synthesis		Leukocyte	=
Cysteine uptake		Leukocyte	M < F
Others non radical markers:			
CuZnSOD	Plasma (2)	Erythrocyte	=
^f^ Peroxides	Plasma; Urine	Placenta	M > F

A.F.: Airway fluid, includes Bronchoalveolar lavage lavage or fluid/tracheobronchial aspirate fluid /airway aspirates fluid; FRAP: Ferric reducing ability of plasma; GPx: glutathione peroxidase; GSSG-R: Disulfide glutathione reductase; GST: Glutatione S-transferase; γ-GT: gamma-glutamyltranspeptidase; MDA: malondialdehyde; 8-OHdG: 8-hydroydeoxy-guanosine; SOD: superoxide dismutase; TAA: Total antioxidant activity; TAC: Total antioxidant capacity; TAOC: Total antioxidant capacity; TBARS: thiobarbituric acid reactive substance; TOS: Total oxidant status; ^a^ All-time, “Plasma” could include “serum”; ^b^ The number in parentheses indicates the number of studies when more than one; ^c^ =: no difference between F and M; ^d^ Umbilical cord vein infused with *tert*-butylhydroxyperoxide, efflux of glutathione; ^e^ “Erythrocyte” can include “red blood cell” and “whole blood”; ^f^ “Peroxide” could include “hydroperoxide” or “total peroxide”.
